# The Association between HbA1c and Cardiovascular Disease Markers in a Remote Indigenous Australian Community with and without Diagnosed Diabetes

**DOI:** 10.1155/2016/5342304

**Published:** 2016-02-17

**Authors:** Luke W. Arnold, Wendy E. Hoy, Suresh K. Sharma, Zhiqiang Wang

**Affiliations:** ^1^Centre for Chronic Disease, The University of Queensland School of Medicine, Royal Brisbane & Women's Hospital, Health Sciences Building Level 8, Herston, QLD 4029, Australia; ^2^Division of Medicine, Royal Darwin Hospital, Top End Health Network, Northern Territory Government, Tiwi, NT 0810, Australia

## Abstract

*Objectives*. This study investigates the burden of cardiovascular risk markers in people with and without diabetes in a remote Indigenous Australian community, based on their HbA1c concentration.* Methods*. This study included health screening exams of 1187 remote Indigenous residents over 15 years old who represented 70% of the age-eligible community. The participants were stratified by HbA1c into 5 groups using cut-off points recommended by international organisations. The associations of traditional cardiovascular risk markers with HbA1c groups were assessed using logistic and linear regressions and ANOVA models.* Results*. Of the 1187 participants, 158 (13%) had a previous diabetes diagnosis, up to 568 (48%) were at high risk (5.7–6.4% (39–46 mmol/mol) HbA1c), and 67 (6%) potential new cases of diabetes (≥6.5% (48 mmol/mol)) were identified. Individuals with higher HbA1c levels were more likely to have albuminuria (OR 3.14, 95% CI 1.26–7.82) and dyslipidaemia (OR 2.37, 95% CI 1.29–4.34) and visited the clinic more often (OR 2.52, 95% CI 1.26–4.99). Almost all traditional CVD risk factors showed a positive association with HbA1c.* Conclusions*. Screening in this remote Indigenous Australian community highlights the high proportion of individuals who are at high risk of diabetes as indicated by HbA1c and who also had an accentuated cardiovascular risk profile.

## 1. Introduction

The Indigenous population of Australia are 3 times more likely to have type 2 diabetes than their non-Indigenous counterparts, have an earlier onset of diabetes, and experience higher rates of diabetes complications [1, 2]. This disparity can also vary significantly among Indigenous communities. Some Indigenous communities are up to 10 times more likely to have diabetes than the general population and 2 times more than other remote Indigenous communities [3, 4]. This further disadvantage for individuals living in remote communities requires continued efforts.

Many previous studies report the burden of diabetes based on diabetes incidence, prevalence, or microvascular and macrovascular complications associated with diabetes [5–7]. It is also important to investigate the burden of this disease in remote Indigenous Australians before they are diagnosed with diabetes. The risk of, and progression of, type 2 diabetes is on a continuum, so the earliest identification of these high-risk individuals would increase the chance to delay or prevent diabetes development.

Use of glycosylated haemoglobin (HbA1c) may be a successful screening option to investigate the burden of diabetes in these remote Indigenous Australian communities. HbA1c is currently recommended for diabetes diagnosis in clinical practice in the United Kingdom, New Zealand, United States, and Australian [8–11] general populations. HbA1c has also been shown to successfully screen urban Indigenous Australians [12] and more recently remote Indigenous Australian communities for diabetes [13].

In addition to diagnosing diabetes, HbA1c could also identify individuals at high risk of developing diabetes. An International Expert Committee recommended a 6.0–6.4% (42–46 mmol/mol) HbA1c category to identify those at high risk of diabetes [14], while the American Diabetes Association (ADA) recommends a 5.7–6.4% (39–46 mmol/mol) HbA1c category [9]. Although diabetes risk is a continuum, these categories are a starting point for health services to focus on prevention and early identification of those at high risk of diabetes. Although high-risk categories have been recommended in general populations, it is unknown what level of HbA1c would be most appropriate in the Indigenous Australian context.

This study aims to investigate the cardiovascular risk profile of people in a remote Indigenous Australian community across a wide range of HbA1c levels. We also compare the differences between groups with and without diabetes to highlight how the burden of cardiovascular risk markers changes as risk of diabetes increases based on increased HbA1c.

## 2. Methods

### 2.1. Participants

Health screening examinations were offered between 2004 and 2006 to every individual of a remote Indigenous Australian community in the Northern Territory who were ambulatory. People in old age care, in hospital, on dialysis, resident elsewhere, or out of community on the screening occasion did not participate. All participants provided written informed consent. Blood and urine samples were collected, anthropometric measurements and lifestyle questions were recorded, and medical records were reviewed. Blood samples were only taken from participants over 15 years old. There were 1,554 who participated in the health screening examinations and 79 percent were aged between 15 and 82 years, which represented 70% of the (1,757) total age-eligible population, based on the 2006 Census. Participants were excluded from the current analysis if they did not identify themselves as Indigenous Australian or did not have a blood sample taken.

### 2.2. Measurements and Identification of Events

Urine and blood samples collected from participants were sent to Westerns Pathology in Darwin. All assays were performed using the Roche Integra I800. Urinary albumin was measured by immunoturbidimetry using the Beckmann Array (Beckman Instruments, Brea, CA). Urinary creatinine was measured using the kinetic method with an alkaline picrate reagent. Samples were analysed within 48 hours of collection, with interim storage at 4°C. Microalbuminuria and macroalbuminuria were classified by a urinary albumin/creatinine ratio (ACR) of 3.4–33 g/mol and ≥34 g/mol, respectively [15]. Blood pressure was measured on the right arm in a seated position after participants had rested for at least 5 minutes using an automated BP device (Welch Allyn, Skaneateles Falls, NY) and appropriate cuffs for arm size. Hypertension was defined if the participant had a previous diagnosis of hypertension in their health records, was taking antihypertensive medication, or had elevated levels in the baseline examination (≥140 mmHg systolic, ≥90 mmHg diastolic). Estimated glomerular filtration rate (eGFR) was calculated using the CKD-EPI (Chronic Kidney Disease Epidemiology Collaboration) formula as validated (without adjustment for African American status) in Indigenous Australian populations by Maple-Brown et al. [16]. Triglyceride levels were measured using lipase/glycerol kinase/GPO-PAP assays. Total cholesterol concentration was measured using cholesterol oxidase/peroxidase reagents. HDL cholesterol levels were measured using cyclodextrin sulphate/PEG modified enzymes. Dyslipidaemia was defined by a previous diagnosis of high cholesterol, taking cholesterol management medication, or having total cholesterol ≥ 5.5 mmol/L or HDL < 1.0 mmol/L at the screening examination as per the Australian Diabetes, Obesity, and Lifestyle Study (AusDiab) [17]. High sensitivity CRP was analysed using immunoturbidimetric CRP assay on a Hitachi 917 analyser (Roche Diagnostics, Australia) with a detection limit of 0.03 mg/L. The assay's analytical range was from 0.1 to 20 mg/L. CRP concentrations greater than 20 mg/L were measured using diluted samples. The imprecision of the assay is less than 5% [18]. Waist circumference (WC) was measured at the narrowest point below the ribs or halfway between the lower border of the ribs and the iliac crest in centimetres. Participants in the highest quartile for WC based on age- and sex-adjusted* z*-scores were defined as having high WC. Body mass index (BMI) was calculated by dividing the individual's weight in kilograms by their height in metres-squared (kg/m^2^). Smoking status was self-reported in the health screening interview. The number of clinic visits in the last year was counted through the clinic records for each individual in the past 12 months prior to their health screening examination. A history of CVD was determined if the participant had hospitalisation associated with CVD in the last 12 years prior to their health screening examination. The number of cardiovascular risk factors was the total score of the following possible risk factors: micro/macroalbuminuria, hypertension, dyslipidaemia, current smoking, and high WC.

### 2.3. Statistical Analysis

The study population was stratified by HbA1c into 5 groups of participants, as follows: four groups without a prior diagnosis of diabetes (<5.7% (39 mmol/mol), 5.7–5.9% (39–41 mmol/mol), 6.0–6.4% (42–46 mmol/mol), and ≥6.5% (48 mmol/mol)) and a single group of participants with known diabetes (4.9–15.6% (30–147 mmol/mol)). The HbA1c categories were created according to the recommended definitions of high risk of diabetes and diabetes diagnosis from the World Health Organization (≥6.5% (48 mmol/mol) HbA1c diabetes diagnosis), the International Expert Committee (IEC) (6.0–6.4% (42–46 mmol/mol) HbA1c for high risk of diabetes), and the American Diabetes Association (ADA) (5.7–6.4% (39–46 mmol/mol) HbA1c for high risk of diabetes) [9, 14, 19]. The number of clinic visits was categorised into low (<5), moderate (5–15), and high (>15) for analysis. Age- and sex-adjusted means and 95% confidence intervals of traditional chronic disease risk factors were calculated for the categories of HbA1c and the differences were tested using linear regression and two-way ANOVA. Logistic regression tested the trend and calculated odds ratios among HbA1c categories for dichotomous and categorical variables. All analyses were conducted using Stata 13.0 [20].

## 3. Results

A total of 1187 participants over 15 years old with HbA1c measurements were included in the analysis. Sixty-seven (6%) had HbA1c greater than or equal to 6.5% (48 mmol/mol) but had no record of diagnosis or management of diabetes, while 158 (13%) participants had a prior diagnosis of diabetes. A further 195 participants were identified as being at high risk of developing type 2 diabetes using the IEC (International Expert Committee) 6.0% (42 mmol/mol) cut-off and up to 568 (48%) participants by the ADA criteria of 5.7–6.4% (39–46 mmol/mol). Participants with known diabetes had HbA1c levels at time of screening between 4.9% (30 mmol/mol) and 15.6% (147 mmol/mol). Sixty of the 158 participants with known diabetes (38%) had an HbA1c level between 6.5% (48 mmol/mol) and 8.5% (69 mmol/mol), and 133 (84%) of known diabetes patients were taking hypoglycaemic medication. Eighty-seven participants with known diabetes (55%) had an HbA1c level ≥7.5% (59 mmol/mol).

**Figure 1:**
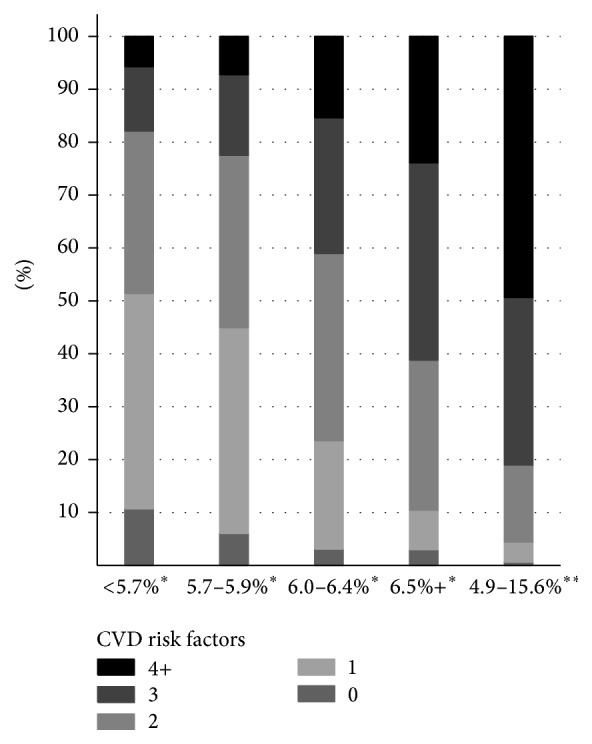
The number of cardiovascular risk factors identified in participants with and without a prior diabetes diagnosis by HbA1c categories. The number of cardiovascular risk factors was the sum of the following possible risk factors: micro/macroalbuminuria, hypertension, dyslipidaemia, current smoking, and high WC. ^*∗*^Participants without a prior diagnosis of diabetes. ^*∗∗*^Participants with a known diabetes diagnosis.

Table 1 details the demographics of the total population over 15 years old in this remote Indigenous community. About 54% of our study participants were men and there was high prevalence of smoking (72%), microalbuminuria (29%), macroalbuminuria (15%), hypertension (28%), and dyslipidaemia (45%) in this community. Sixty-two participants (5.2%) had a cardiovascular event prior to their health examination.  Table 2 shows the baseline characteristics by HbA1c categories. As expected, those at higher risk of diabetes tended to be older. Women made up a larger proportion of the higher HbA1c groups and a lower proportion of the lower HbA1c groups. There was a direct relationship of categories of HbA1c with several cardiovascular risk markers, such as systolic blood pressure, body size, triglycerides, C-reactive protein, and an inverse association with HDL cholesterol levels. The prevalence of microalbuminuria, macroalbuminuria, dyslipidaemia, and hypertension was also higher in groups with higher HbA1c. A larger proportion of participants from the higher HbA1c categories visited the local clinic a high or moderate number of times compared to the lowest HbA1c group. Odds ratios in Table 3 also show that participants in the higher HbA1c groups were more likely to show indicators of risk than those in the lowest HbA1c % group.  Figure 1 highlights that participants with higher HbA1c had higher numbers of cardiovascular disease risk factors. Over 75% of the participants in the 6.0–6.4% (42–46 mmol/mol) HbA1c group had 2 or more cardiovascular risk factors, compared with 90% for participants in the ≥6.5% (48 mmol/mol) HbA1c group. Over 95% of participants with known diabetes had 2 or more cardiovascular risk factors, with nearly half (49%) having 4 or more risk factors.

## 4. Discussion

The screening results of glycosylated haemoglobin levels in this remote Indigenous Australian community highlight the significant burden of diabetes, risk of diabetes, and cardiovascular risk factors force on this population. HbA1c screening of people not previously diagnosed with diabetes identified a significant proportion (6%) of people living with HbA1c levels over the cut-off recommendations for diabetes diagnosis of ≥6.5% (48 mmol/mol) recommended by WHO and Marley et al. [13, 19]. Over half of all nondiabetic participants were at high risk of developing diabetes according to the ADA specifications (5.7–6.4% (39–46 mmol/mol) HbA1c) [9]. Participants in the higher HbA1c categories had the highest rates of albuminuria, dyslipidaemia, and hypertension and had the highest number of cardiovascular risk factors.

To our knowledge this is the first and largest community-wide HbA1c screen conducted in remote Indigenous Australians. It is also the first demonstration of an association of HbA1c with cardiovascular risk factors in nondiabetic Indigenous Australians. We have shown that almost half (44%) of this community over 15 years old, based on the 2006 Census population estimate, either had diabetes or was at high risk of developing diabetes and with a significant number of comorbid cardiovascular risk factors. This proportion is similar to that observed in high-risk African American and Latino populations in the USA [21]. Our observed burden of diabetes is dramatically higher than the hospital burden, burden of diabetes complications, and incidence/prevalence rates of diabetes indicated in Aboriginal Australia [5–7]. Such a high proportion of Indigenous Australians living with a high risk of diabetes and other coexisting CVD risk factors presents a substantial challenge.

This study has identified that a large number of participants in this remote community (29%) were only captured as being at high risk of diabetes using the ADA cut-off point of 5.7–5.9%. Over half of this group had 2 or more cardiovascular risk factors as shown in Figure 1; however the odds ratios in Table 3 suggest their HbA1c level did not elevate their risk of CVD risk markers. The ADA guidelines used the cut-off of 5.7% due to its high positive predictive value (equivalent to 100 mg/dL fasting glucose) to identify people at high risk of later development of diabetes and the effective prevention of diabetes in people targeted between 5.5 and 6.0%, according to the Diabetes Prevention Program (DPP) [9]. In the Indigenous Australian context, this study confirms there are a significant proportion of participants who may benefit from diabetes prevention initiatives at this point of the diabetes risk continuum. Interventions to control the limited existing cardiovascular risk markers in this group may provide the best opportunity to identify high-risk individuals and prevent the development of diabetes in remote Indigenous populations such as this.

The findings of this study also suggest an increased level of systemic inflammation as risk of diabetes increases, indicated by higher levels of circulating C-reactive protein in the higher HbA1c categories. This is consistent with previous findings suggesting a potential role of systemic inflammation in insulin resistance and glucose intolerance [22, 23]. Wu et al. suspected hyperreaction of the innate immune system as a potential explanation for their observed association of elevated CRP with higher HbA1c but could not confirm this mechanism [23]. The present study did not investigate the underlying mechanism of the association but supports further investigation of this potential role within the Indigenous Australian population.

These findings suggest that HbA1c screening might identify those at high risk of diabetes in many other urban and remote Indigenous Australian communities. The World Health Organization recognises the strength of HbA1c in screening for diabetes; however it suggests a worldwide rollout of the screening is largely limited by the resource difficulties for developing nations to establish standardised practice [19]. This study recognises Australia as a developed nation with the resources to conduct relative cost-effective and standardised HbA1c screening in high-risk populations; however a systematic cost-benefit analysis is yet to be undertaken [24]. Early detection, delayed progression, and prevention of diabetes are high priority areas in Australia and this study has identified HbA1c screening as a useful tool in this remote Indigenous Australian community.

The introduction of item 715 (Medicare Health Assessment for Aboriginal and Torres Strait Islander People) into the Medicare Benefits Schedule (MBS) by the Australian government was designed to encourage regular repeated testing of Aboriginal adults for the early detection and intervention for conditions that cause morbidity and early mortality [25], and it is applied with increasing frequency and regularity in many Indigenous health care settings. The findings of this study encourage the movement towards incorporating HbA1c screening as part of these health assessments [19]. Furthermore, in November 2014, funding for laboratory HbA1c testing was included in the MBS for individuals at high risk of diabetes, which significantly increases the opportunity for clinicians to monitor HbA1c in this setting [26]. HbA1c can be measured from a standard blood sample without the need for patient fasting or consumption of glucose loads, making it a much more convenient test than oral glucose tolerance and fasting plasma glucose tests [8].

The findings of this study suggest HbA1c may be useful in detecting risk of diabetes and cardiovascular risk factors in the primary health care setting. This evidence encourages the adoption of HbA1c screening as an effective initiative to monitor risk of diabetes. Further research is needed, however, to assess the specific clinical HbA1c cut-off points for diagnosis of diabetes in remote Indigenous Australians. This study also confirms the need for repeated testing throughout life, to detect cases of diabetes or prediabetes as they emerge with increasing age [4].

The high number of clinic visits in the higher HbA1c groups can be interpreted in two ways. It may suggest that those at high risk of diabetes and with a high number of cardiovascular risk factors are accessing the medical treatment that they require and are under consistent monitoring, which is very positive from a perspective of access to health care for high-risk populations [27]. The high clinic visits could also be interpreted in a way that suggests individuals with high risk of diabetes and cardiovascular disease are a significant burden on the health care system in these remote Indigenous communities, which should be addressed. If high numbers of clinic visits in these remote communities are related to chronic disease risk factors, there may be an opportunity to relieve the primary health care system by targeting health promotion initiatives at early prevention of chronic diseases.

There are limitations of this study which must be acknowledged before generalising these findings to other populations. The wide confidence intervals surrounding the odd ratios reported in Table 3 suggest a high level of uncertainty of the association. There were difficulties interpreting the data due to the significant variation of age and gender amongst the HbA1c groups and small sample size. We acknowledge that HbA1c may not be suitable to independently predict microalbuminuria, macroalbuminuria, dyslipidaemia, and hypertension and that it has not yet been evaluated for this role. However, this study's findings suggest utilising HbA1c to screen individuals for diabetes and risk of diabetes is a suitable test which captures a high proportion of individuals who have these comorbidities and cardiovascular risk factors. This study recognises that diabetes development occurs on a continuum and that categories for high risk of diabetes do not necessarily represent an exact point of higher clinical risk to diabetes. The HbA1c groups simply allow a more convenient way to categorise the population for health promotion interventions. Another limitation of this study is that we did not have information on fasting plasma glucose or 2 h oral glucose tolerance tests to determine the sensitivity, specificity, and ROC curves of HbA1c screening compared to these previous methods. The use of HbA1c is warranted in this setting as a previous study found that HbA1c was significantly more likely to determine a diabetes diagnosis than blood glucose tests in other remote Indigenous communities [28]. Finally, we had to rely on existing literature to guide our HbA1c definitions of diabetes and high risk of diabetes.

## 5. Conclusion

Glycosylated haemoglobin screening of people not known to have diabetes in this remote Indigenous Australian community identified a high proportion of the population at high risk of developing or with diabetes using definitions in the literature. HbA1c screening in Australian remote communities is a convenient blood test which has the ability to detect individuals at high risk of diabetes earlier and who may already be suffering from a significant number of comorbid cardiovascular risk factors.

## Figures and Tables

**Table 1:** Characteristics of the total population over 15 years old of a remote Indigenous Australian community.

	Total population
*n* (%)	1187
Age	33 (24–44)
Men, %	54
HbA1c, %	5.8 (5.5–6.1)
SBP, mmHg	118 (108–129)
Cholesterol, mmol/L	4.7 (4–5.3)
HDL chol, mmol/L	1.2 (1.1–1.5)
LDL chol, mmol/L	2.55 (2.0–3.1)
BMI, kg/m^2^	22.7 (19.5–27.2)
Waist, cm	87 (77–97.5)
ACR, g/mol^b^	2.58 (0.95–11.25)
Triglycerides, mmol/L^b^	1.5 (1.0–2.4)
CRP, mg/L^b^	6 (2–11)
eGFR, mL/min/1.73 m^2^ ^b^	108.9 (95.5–121.5)
Current smoker, %	72
Microalbuminuria, %^c^	29
Macroalbuminuria, %^d^	15
Hypertension, %^e^	28
Dyslipidaemia, %^f^	45
Number of clinic visits in the previous 12 months	
High (>15)^a^	17 (204)
Moderate (5–15)^a^	39 (463)
Low (<5)^a^	43 (513)
CVD history^a^	5.2 (62)

Median (25th–75th percentiles) unless otherwise stated. SBP, systolic blood pressure; HDL, high-density lipoproteins; LDL, low-density lipoproteins; BMI, body mass index; ACR, albumin/creatinine ratio; CRP, C-reactive protein; eGFR, estimated glomerular filtration rate.

^a^% (*n*).

^b^Geometric mean (95% CI).

^c^ACR 3.4–33 g/mol.

^d^ACR ≥ 34 g/mol.

^e^Dyslipidaemia was defined by a previous diagnosis of high cholesterol, taking cholesterol management medication, or having total cholesterol ≥ 5.5 mmol/L or HDL < 1.0 mmol/L.

^f^Hypertension was defined if the participant had a previous diagnosis of hypertension in their health records, was taking antihypertensive medication, or had elevated levels in the baseline examination (≥140 mmHg systolic, ≥90 mmHg diastolic).

**Table 2:** Characteristics of a remote Indigenous Australian community over 15 years old stratified by HbA1c categories.

	Without a prior diabetes diagnosis				With a prior diagnosis of diabetes	*p* value
	<5.7%	5.7–5.9%	6.0–6.4%	≥6.5%	4.9–15.6%	
*n* (%)	420	347	195	67	158	
Age	27 (21–34)	31 (23–39)	39 (29–47)	39 (32–53)	48 (40–55)	<0.001
Men, %	64	59	48	42	34	<0.001
HbA1c, %	5.5 (5.3–5.6)	5.8 (5.7–5.9)	6.1 (6.0–6.2)	6.9 (6.7–7.8)	8.0 (6.6–9.8)	<0.001
SBP, mmHg	115 (105–125)	118 (107–128)	120 (109–132)	123 (112–133)	123 (112–141)	<0.001
Cholesterol, mmol/L	4.6 (3.9–5.2)	4.7 (4.1–5.4)	4.7 (4.0–5.5)	4.9 (4.1–5.9)	4.7 (4.0–5.4)	0.569
HDL chol, mmol/L	1.3 (1.1–1.5)	1.3 (1.1–1.5)	1.2 (1.0–1.5)	1.1 (0.9–1.3)	1.2 (1.0–1.3)	<0.001
LDL chol, mmol/L	2.5 (2.0–3.1)	2.6 (2.1–3.2)	2.6 (2.0–3.2)	2.5 (1.8–3.2)	2.2 (1.8–3.0)	0.213
BMI, kg/m^2^	20.5 (18.6–24.8)	21.2 (19.0–25.0)	24.2 (21.2–28.6)	27.1 (23.0–31.7)	27.5 (23.0–31.7)	<0.001
Waist, cm	81 (73–90)	83 (74.4–93)	93 (83–102)	99 (92–109)	102 (93–111.3)	<0.001
ACR, g/mol^b^	1.37 (0.8–5.0)	1.74 (0.8–6.3)	4.50 (1.5–17.7)	11.68 (2.3–48.3)	31.29 (5.8–101.9)	<0.001
Triglycerides, mmol/L^b^	1.2 (0.9–1.8)	1.3 (0.9–2.0)	1.7 (1.3–2.5)	2.8 (2.0–4.0)	2.5 (1.7–3.3)	<0.001
CRP, mg/L^b^	4 (2–7)	5 (2–9)	7 (4–13)	10 (5–17)	10 (6–16)	<0.001
eGFR, mL/min/1.73 m^2^ ^b^	114.2 (102–124)	112.5 (100–122)	104.8 (91–118)	105.5 (92–117)	94.3 (82–109)	0.900
Current smoker, %	74	74	70	70	64	0.140
Microalbuminuria, %^c^	23	27	38	40	34	<0.0001
Macroalbuminuria, %^d^	6	7	19	27	47	<0.0001
Hypertension, %^e^	15	20	34	40	67	<0.0001
Dyslipidaemia, %^f^	30	37	57	69	80	<0.0001
Number of clinic visits in the previous 12 months						
High (>15)^a^	10.2 (43)	14.4 (50)	21.5 (42)	20.9 (14)	34.8 (55)	<0.0001
Moderate (5–15)^a^	35.7 (150)	34.5 (130)	41.5 (81)	52.2 (35)	42.4 (67)	<0.0001
Low (<5)^a^	54.0 (227)	48.1 (167)	36.9 (72)	26.9 (18)	22.8 (36)	<0.0001
CVD history^a^	2.9 (12)	2.6 (9)	8.2 (16)	9.0 (6)	12.0 (19)	<0.0001

Median (25th–75th percentiles) unless otherwise stated. SBP, systolic blood pressure; HDL, high-density lipoproteins; LDL, low-density lipoproteins; BMI, body mass index; ACR, albumin/creatinine ratio; CRP, C-reactive protein; eGFR, estimated glomerular filtration rate.

^a^% (*n*).

^b^Geometric mean (95% CI).

^c^ACR 3.4–33 g/mol.

^d^ACR ≥ 34 g/mol.

^e^Dyslipidaemia was defined by a previous diagnosis of high cholesterol, taking cholesterol management medication, or having total cholesterol ≥ 5.5 mmol/L or HDL < 1.0 mmol/L.

^f^Hypertension was defined if the participant had a previous diagnosis of hypertension in their health records, was taking antihypertensive medication, or had elevated levels in the baseline examination (≥140 mmHg systolic, ≥90 mmHg diastolic).

**Table 3:** Odds ratios (OR) of comorbidities for participants with and without a prior diabetes diagnosis stratified by HbA1c% categories.

	*n*	Without a prior diabetes diagnosis							With a prior diagnosis of diabetes	
		<5.7	5.7–5.9		6.0–6.4		≥6.5		4.9–15.6	
		OR	OR	95% CI	OR	95% CI	OR	95% CI	OR	95% CI
Microalbuminuria^d^										
Unadjusted	989	1	1.31	(0.94–1.82)	2.65	(1.80–3.91)	4.28	(2.28–8.03)	5.81	(3.48–9.68)
Age and sex adjusted	989	1	1.10	(0.78–1.55)	1.73	(1.14–2.62)	2.28	(1.16–4.47)	2.22	(1.25–3.95)
Final model^a^	981	1	1.06	(0.75–1.51)	1.53	(0.99–2.34)	1.89	(0.94–3.80)	1.76	(0.95–3.26)
Macroalbuminuria^d^										
Unadjusted	823	1	1.23	(0.70–2.19)	4.76	(2.74–8.28)	10.25	(4.81–21.81)	28.58	(15.89–51.41)
Age and sex adjusted	823	1	0.91	(0.50–1.65)	2.81	(1.55–5.09)	5.07	(2.25–11.41)	10.27	(5.30–19.93)
Final model^a^	814	1	1.01	(0.53–1.90)	1.97	(1.02–3.83)	3.14	(1.26–7.82)	6.05	(2.86–12.77)
Hypertension^e^										
Unadjusted	1187	1	1.43	(0.98–2.08)	2.97	(1.99–4.42)	3.83	(2.19–6.68)	11.55	(7.54–17.69)
Age and sex adjusted	1187	1	1.14	(0.76–1.71)	1.79	(1.14–2.81)	1.82	(0.96–3.45)	4.84	(2.92–8.03)
Final model^b^	1159	1	1.12	(0.73–1.71)	1.34	(0.82–2.17)	0.97	(0.48–1.95)	2.86	(1.66–4.95)
Dyslipidaemia^f^										
Unadjusted	1187	1	1.36	(1.01–1.84)	3.08	(2.17–4.38)	5.11	(2.93–8.92)	9.19	(5.91–14.27)
Age and sex adjusted	1187	1	1.22	(0.90–1.67)	2.38	(1.64–3.46)	3.51	(1.95–6.30)	5.27	(3.23–8.58)
Final model^c^	1183	1	1.21	(0.88–1.66)	1.97	(1.34–2.90)	2.37	(1.29–4.34)	3.34	(1.99–5.60)
Number of clinic visits in the previous 12 months										
High (>15)^g^										
Unadjusted	717	1	1.58	(1.00–2.48)	3.10	(1.87–5.11)	4.07	(1.88–8.80)	8.72	(5.08–14.98)
Age and sex adjusted	717	1	1.35	(0.84–2.17)	1.94	(1.12–3.35)	1.94	(0.84–4.48)	3.48	(1.86–6.49)
Final model^a^	705	1	1.52	(0.94–2.46)	1.83	(1.04–3.24)	2.25	(0.91–5.59)	2.47	(1.25–4.88)
Moderate (5–15)^g^										
Unadjusted	976	1	1.17	(0.86–1.60)	1.71	(1.17–2.50)	2.92	(1.59–5.34)	3.05	(1.91–4.85)
Age and sex adjusted	976	1	1.10	(0.80–1.50)	1.42	(0.96–2.12)	2.10	(1.11–3.95)	1.95	(1.16–3.30)
Final model^a^	965	1	1.13	(0.82–1.55)	1.42	(0.95–2.13)	2.52	(1.26–4.99)	1.96	(1.12–3.41)

OR, odds ratio.

^a^Age, sex, systolic blood pressure, waist circumference, total cholesterol, HDL cholesterol, current smoking status, and hypertensive medication were included in the final model.

^b^Age, sex, waist circumference, total cholesterol, HDL cholesterol, and current smoking status were included in the final model.

^c^Age, sex, systolic blood pressure, waist circumference, current smoking status, and hypertensive medication were included in the final model.

^d^Compared with participants with normal ACR levels (<3.4).

^e^Hypertension was defined if the participant had a previous diagnosis of hypertension in their health records, was taking antihypertensive medication, or had elevated levels in the baseline examination (≥140 mmHg systolic, ≥90 mmHg diastolic).

^f^Dyslipidaemia was defined by a previous diagnosis of high cholesterol, taking high cholesterol management medication, or having total cholesterol ≥ 5.5 mmol/L or HDL < 1.0 mmol/L.

^g^Compared with participants with low number of clinic visits in the previous 12 months (<5).

## Abbreviations

ACR: Albumin/creatinine ratio
ADA: American Diabetes Association
AusDiab: Australian Diabetes, Obesity, and Lifestyle Study
BMI: Body mass index
CI: 95% Confidence interval
CKD-EPI: Chronic Kidney Disease Epidemiology Collaboration
CVD: Cardiovascular disease
DPP: Diabetes Prevention Program
eGFR: Estimated glomerular filtration rate
HbA1c: Glycosylated haemoglobin
MDRD: Modification of Diet in Renal Disease
OR: Odds ratio
T2D: Type 2 diabetes
WC: Waist circumference
WHO: World Health Organization.
